# A qualitative study of parental caregiving experience in adolescents with depression from the perspective of positive psychology in China

**DOI:** 10.3389/fpubh.2025.1454085

**Published:** 2025-01-29

**Authors:** Yueyan Zhao, Qunfang Miao, Siying Xin, Peiqing Li, Yaoyao Huang

**Affiliations:** ^1^School of Nursing, Hangzhou Normal University, Hangzhou City, Zhejiang Province, China; ^2^School of Clinical Medicine, Hangzhou Normal University, Hangzhou City, Zhejiang Province, China

**Keywords:** adolescents, depression, parents, caring experience, qualitative study

## Abstract

**Objective:**

To explore the inner experiences and needs of parents of adolescents with depression in the caregiving process, to understand the problems they encounter, and to provide precise intervention and support strategies for this group from a positive psychology orientation.

**Methods:**

Use of qualitative research methods, semi-structured interviews were conducted with 12 parents of adolescents with depression, selected from December 2022 to October 2023. Colaizzi′s seven-step analysis method was used to extract themes.

**Results:**

The parental caregiving experience for adolescents with depression can be grouped into 3 themes and 8 sub-themes: ① Complex negative emotions (negative emotions at the beginning of diagnosis, negative emotions during the caregiving process); ② Adversity experienced by individuals and families (increased physical and financial burdens, lack of access to professional support information, family dysfunction); ③ Coping and adaptation to adversity (self-growth, actively seeking outside assistance, enhanced family connection).

**Conclusion:**

Parents of adolescents with depression have complex caregiving experiences and face numerous difficulties and challenges at different stages of treatment. Caregivers and medical social workers should focus on the inner experiences and needs of parents at various stages of caregiving. Establishment of a joint care model with a positive psychology perspective to enhance individual and family psychological resilience and a sense of disease benefit.

## Introduction

Depression is a common clinical mental illness characterized by high prevalence, high relapse rate, high suicide rate, and high disability rate (1). Studies have shown that the prevalence of depression among children and adolescents in China is 3.2%, the highest level in recent years (2). Depression significantly impacts adolescents’ mental and physical health, academic performance, and social functioning, potentially leading to adverse events such as self-harm and suicide (3, 4). In the absence of health resources and social support, parents, as the primary family caregivers of adolescents with depression, must not only monitor changes in the adolescents’ depression and prevent extreme events but also face significant social pressure and physical and economic burdens (5–7). Studies show that the mental health literacy of parents of adolescents with depression is low (8). In recent years, positive psychology has emerged as a research hotspot, highlighting that positive changes in caregivers’ emotions and behaviors can enable them to adapt positively to caregiving stress and improve their quality of life (9). Given the heavy caregiving burden of parents with depressed adolescents, it is crucial to understand how positive psychology can transform this burden into positive emotions and behaviors. Therefore, this study adopts a qualitative approach to explore the changes in the inner experiences and needs of parents of adolescents with depression during caregiving from the perspective of positive psychology, providing evidence to support the development of interventions and support strategies for this group.

## Methods

### Design and sample

Using purposive sampling, Parents of adolescent depression patients who attended a joint outpatient clinic for child and adolescent psychology in a tertiary-level hospital in Zhejiang Province from December 2022 to October 2023 were selected for the study. Inclusion criteria: ① fathers or mothers of adolescent depression patients aged 12–18 years old; ② being the main caregivers of the patients; ③ informed consent and voluntary participation; ④ being able to express thoughts clearly in words. Exclusion criteria: ① suffering from mental disorders and other diseases; ② having a clear diagnosis of severe chronic physical diseases.

### Participant characteristics

Purposive sampling was used, and the decision of the study sample size was based on the criterion of information saturation (10). A total of 12 parents of adolescents with depression were ultimately selected for interviews. To protect the interviewees’ privacy, the interviewees were replaced by the numbers C1-C12.Detailed demographic data are presented in Table 1.

**Table 1:** General information on adolescents with depression and their parents.

Number	Gender	Age (years)	Education level	Relationship with patient	Marital status	Patient age (years)	Duration of patient’s illness
C1	Female	38	University	Mother-son	Married	15	6 months
C2	Female	41	High School	Mother-son	Married	14	1 year and 3 months
C3	Female	40	University	Mother-daughter	Married	16	8 months
C4	Female	50	Primary School	Mother-son	Married	16	2 years
C5	Female	46	Junior High School	Mother-daughter	Married	16	1 year and 6 months
C6	Female	38	University	Mother-daughter	Married	15	5 months
C7	Female	45	Junior High School	Mother-daughter	Married	18	4 years
C8	Female	39	Junior College	Mother-son	Divorced	15	2 years and 7 months
C9	Female	42	High School	Mother-son	Married	18	11 months
C10	Male	42	University	Father-daughter	Married	15	1 year
C11	Female	39	High School	Mother-daughter	Married	14	7 months
C12	Male	41	Junior High School	Mother-son	Married	17	2 years and 3 months

### Interview outline

Determining the Interview Outline The semi-structured interview method was adopted, centering on the caregiving experience of parents of adolescents with depression. After reviewing relevant literature and consulting three psychologists in the field as well as a senior chief nurse, the interview outline was initially drafted. During the interviews, parents of adolescents with depression in treatment were guided to review their caregiving experiences during the diagnostic and treatment interventions to answer the questions in the interview outline. After pre-interviewing the parents of two adolescents with depression, the outline was revised based on the results of the interviews to finalize the interview outline. The outline included the following questions: (1) What were your thoughts and feelings during the different stages of your child’s illness? How did you cope? (2) Do you think there were any changes in yourself and your family during the caregiving process? (3) What do you think prompted these changes in you and your family? (4) What impact did these changes have on you and your family? (5) What kind of growth or gain did you and your family experience throughout the caregiving process?

### Data collection methods

The research team consisted of nursing postgraduate students and an associate professor both of whom have extensive experience in conducting qualitative research studies. The personal interview method was used to collect data from parents of adolescents with depression. The purpose, content, method, and significance of the study were explained in detail to the research subjects before the start of the interviews, and they were informed that the interviews would be synchronously recorded. Commitment to confidentiality and the principle of non-maleficence was ensured. After the interview subjects signed the informed consent form, each interview was conducted for 30–45 min. During the interview process, the interviewer maintained a neutral attitude without any guidance or hints. The researcher clarified and reviewed any ambiguous content promptly to ensure the authenticity and completeness of the information.

### Data management and analysis

After the interview, the interviewer transcribed, summarized, and collated the interview data within 24 h. It was reviewed and confirmed by another researcher. Data were analyzed independently by 2 researchers using the Colaizzi 7-step analysis method (11). In cases of disagreement, The analysis was returned to the subject group for discussion to reach a consensus.

### Rigor and reflexivity

First, our mentor is a professional psychologist with a wealth of interviewing skills, and our group members have all received training in qualitative research as well as interviewing skills. Second, the interviewer would revert to the interviewee for verification of any questionable or inaccurate information encountered during the interview. Thirdly, all the interview data were returned to the interviewees and it had been ascertained that the content of the interviews were their true thoughts. The authenticity, completeness and accuracy of the data were ensured. Fourthly, the researchers took a neutral stance when coding the data. The data were analysed independently by two researchers. Finally, the final coding and themes were determined through discussions among the members of the research team.

### Ethical considerations

The study was approved by the ethical review committee of the university (Ethical Review Permission No. 20190096).

## Findings

Based on the qualitative data, the parental caregiving experience for adolescents with depression can be grouped into 3 themes and 8 sub-themes.

### Theme 1: complex negative emotions

#### Negative emotions arising in the early stages of diagnosis

① Denial and skepticism. Respondents mostly showed denial and skepticism in the early stages of seeking a medical diagnosis. *C3: “We do not even have depression in our family, I do not believe she’s depressed, she’s just reaching puberty.” C6: “She just goes on the internet more and learns from examples; nowadays, it is popular to be depressed and emo on the internet, so she thinks she is also depressed.”* ② Regret and guilt. In accepting the fact that they were ill, respondents were caught up in self-blame, guilt, and regret for a long period, often blaming themselves entirely for the patient’s illness. *C1: “If only I had quarreled less in front of him or complained less to him, he would not have been so affected.” C7: “If only I had listened to her ‘complaints’ more, I would not have caused her to get this disease. I regret it.”* ③ Anxiety and worry. With the initial knowledge of the disease, many interviewees expressed anxiety and worry about the treatment process and prognosis of depression. *C8: “Now I am just afraid that this disease of his will keep recurring and stay with him for the rest of his life.” C1: “I’m afraid that he will suddenly lose control of his emotions and jump off a building to commit suicide or something.”*

#### Negative emotions arising from the caregiving process

① The sense of unfairness is associated with the mother’s caregiving role. Adolescents with depression who came to the psychological outpatient clinic for treatment were mostly accompanied by their mothers, and some of the mothers expressed feelings of unfair treatment in their caregiving roles during the interviews. *C3: “A woman who cannot raise a child is not a good wife or a good mother. My in-laws and even my mum and dad think that I have not been a good mother and that’s why my child is sick, whereas they are not as critical of my husband.” C4: “Since my child has been sick, everyone thinks that it is because I am not caring enough as a mum for my child, telling me that men are the main focus and women should stay at home, and telling me to give up my career. I am not saying that I am unwilling to focus on my family. What makes me feel resentful is that they have never asked my husband to focus on the family and have never considered that his father and I are equal in terms of our children’s caregiving responsibilities.”* ② Sickness stigma and burnout. During the adolescent’s illness, some respondents reported feeling shame when talking about depression and were reluctant to seek help from outside sources. *C9: “When people ask me why our youngest child is off from school, if he is sick, I am too embarrassed to tell them the truth (that it is because of depression). I feel quite ashamed.” As caregiving time accumulates, patient-parent burnout increases. C7: “Life is stressful nowadays, and I’m so fatigued after work that I just want to be left alone in peace. My husband and I are physically and mentally exhausted from taking care of our child for the past few years.”*

### Theme 2: difficulties encountered by individuals and families

#### Increased physical and financial burden

Interviewees reported that in the “marathon” care tug-of-war, the whole family invested a lot of time and energy in caring for the patient, greatly reducing their time and space, and it cost them a lot of material and financial resources. *C3: “Now every fortnight I have to take leave from my workplace to go to the outpatient clinic, and the rest of the time she’s at school. But as soon as she calls her dad and wants to come home, her dad goes to school to pick her up right away.” C1: “We’ve been to many hospitals, general hospitals, specialist hospitals, and even private clinics, and he does not trust any of the doctors. The registration fee for the psychological outpatient clinic is expensive, and he keeps relapsing with this illness.”*

#### Family dysfunction

There is an increase in verbal and behavioral conflicts between the respondent and the patient in daily life. *C5: “When I say something she does not like, she gets upset right away or even swears.”* In terms of caregiving behaviors, some respondents reported that their children had experienced self-injurious behaviors, so they restricted some of their children’s activities. *C12: “I now keep all the knives in the house. He must answer the phone or be accompanied by me when going out. If he cannot do that, he’s not allowed to go out, and we are always arguing about these things.”* The couple’s relationship is severely tested in the caregiving process. *C6: “I have always accompanied my daughter to the psychiatric clinic. The last time I tried to get her father to come along, he was too ashamed to come, and we had a big fight about it.” C8: “We’ve had several arguments about our child’s subsequent education and life planning.”* Similarly, the kinship relationship within the extended family changed during the caregiving process. *C3: “Before, her uncles and aunts would come to my house for New Year’s and festivals, but since she got sick, they do not visit much anymore.” C4: “Children from relatives who are almost the same age as him do not come to our house to play anymore.”*

#### Lack of access to professional information

Interviewees all reported a lack of channels to acquire disease-related knowledge, with the vast majority of information support coming from hospitals. Medical knowledge obtained through books, the Internet, and other channels lacked personalized guidance. *C12: “There is also a psychological counseling room in his school, but he thinks it is not professional enough. It is difficult to register at public hospitals, and private psychological clinics cannot be trusted. So we do not have a lot of knowledge about diseases.” C10: “For diseases like a cold or stomach disease, the treatment is very clear, even Baidu can show you the information. But depression is too much about personal and family traits; the information found on the Internet does not necessarily apply, and the quality* var*ies. The psychiatric department in hospitals is generally in the provincial capital, which is inconvenient for travel.”*

### Theme 3: hoping to reinvent

#### Self-growth

##### Increased ability to regulate emotions

The interviews revealed that some of the interviewees had experienced distressing emotions surrounding their caregiving, but had slowly improved their emotional regulation through reinterpretation of stressful events and cognitive change, shifting their thinking from a positive perspective. *C3: “I used to wonder, why is this happening to me? The children of my colleagues around me are all psychologically healthy. Then I attended some public lectures at the hospital and read many cases on public websites. I realized that this is quite common and might contribute to the growth of the child and the family.” C2: “My child’s illness made me reflect on my educational style, which feels like both a blessing and a curse.”* Meanwhile, another group of respondents regulated their emotions through convenient and accessible emotional relaxation techniques. *C6: “I heard from my doctor that positive thinking training would help with my emotions. I have positive thinking training apps on my phone and watch, and I use them almost every day.” C8: “When I feel irritable, I usually light aromatherapy candles and listen to soothing music.”*

##### Improved caring skills, increased will to live

Most of the interviewees initially viewed caring as a heavy burden and a great deal of pressure, but over time, through their own positive psychological adjustment, they became more positive in their thinking. They began to focus on their personal growth and were surprised to find that their caring skills improved significantly, their will to live became stronger, and their sense of self-worth increased during the period of caring for others. *C11: “Throughout the treatment, I feel I have improved my skills as a mother. Motherhood may be innate, but caregiving is truly a skill.” C2: “I feel like my resilience has improved, and I have learned about other important aspects of life.”*

##### Actively seeking external assistance

Some of the interviewees have made a gradual transition from passive acceptance of the disease to adapting to the existence of the disease, and then proactively exploring coping strategies to help themselves overcome the adversity by seeking external assistance within their financial means. *C10: “I take the initiative to visit the school psychologist to discuss my child’s situation, sometimes bringing my child along. Firstly, I want to identify the issues between my child and me with the help of professionals. Secondly, I want my child to interact with more teachers at school. Thirdly, because school counseling is free of charge, it alleviates some of our financial burden and fosters a sense of school community.” C1: “I joined a depression support group where we sometimes share tips, drink tea together, relax, and talk about our struggles.”*

##### Enhancement of family connection

① With the treatment of the disease, some respondents’ family values changed, and positive family beliefs were strengthened. *C9: “I used to think that I needed to provide more material security for my child, so I focused on my work. However, now his father and I are gradually refocusing on our family. Providing him with a perfect childhood is now the shared goal of his father and me.” C11: “We believe our family will get better and better and can overcome this big monster together.”* ② As the treatment progresses, family members become more closely connected and share the pressure of caring for the child. *C4: “In the beginning, his father did not take much care of the child, and I was responsible for the child’s upkeep. But now, he helps me take care of the child, takes him out to eat occasionally, and goes to the amusement park. His in-laws come to the house more often, which has significantly reduced the pressure of caring for the child.” C2: “After several counseling sessions, I am slowly able to understand some of my child’s words and talk with him about* var*ious things. The conflicts are not as frequent as before, and even my communication with his father has become much smoother.”*

## Discussion

### Targeted support for different stages of the caring experience

This study found that during the diagnosis-seeking period, most parents of adolescents with depression initially experienced emotions of denial and skepticism, followed by regret and guilt. Subsequently, anxiety and worry became increasingly prominent. Emotions of denial and skepticism are related to a lack of disease awareness; regret and guilt are linked to parents’ beliefs that their children’s illnesses are due to their previous inappropriate communication and education styles and deteriorating spousal relationships (12, 13); anxiety and worry may be connected to concerns about their children’s future development and the parents’ susceptibility traits as first-degree relatives (14). Depression is a chronic disease, and with the accumulation of caregiving time, parental burnout will increase daily. The above suggests that caregivers should provide targeted health education according to the different psychological development characteristics presented by the parents at various stages of the disease to meet their needs effectively. Positive psychology intervention is an intervention model based on the theory of positive psychology, which aims to maximize the exploration and use of people’s own positive potential to improve their positive cognition, emotion or behavior (15). The American Psychological Association defines psychological resilience as the process of adapting in the face of adversity, trauma, tragedy, threat, or other major stressors (16). Depression, as a chronic illness, can be addressed by caregivers through positive psychological interventions throughout the process while providing different disease awareness knowledge depending on the stage of caregiving. Nursing staff can enhance psychological resilience by instructing the parents to engage in positive thinking training and exercise, thus helping them adapt to their roles, reduce fatigue, and mitigate the impact of adverse emotions (17, 18). Additionally, this study found that some mothers felt a clear sense of unfairness during the caregiving stage. This may be related to the shift from traditional gender discourse (mainly referring to the family labor division model of “men work outside, women work inside”) to gender discourse in the era of marketization (e.g., gender equality), where the model of “men work outside, women work inside” has been replaced by “dual-income families” (19, 20). At the same time, studies have shown that women exhibit higher levels of anxiety and depression when facing stressful events, and female caregivers are more likely to suffer from mental health issues (21). The accumulation of these negative emotions poses a significant challenge to the physical and mental health of the patient’s mother. From a nursing perspective, caregivers should pay more attention to the caregiving experiences of female caregivers in the family, guide spouses and other family members to reasonably distribute caregiving responsibilities, reduce the burden on a single caregiver, and help the parents practice mindfulness adjustments. This study found that most respondent families experienced a sense of disease shame during the caregiving process. Disease shame refers to the self-stigmatization of patients with a certain disease, originating from the internalization of negative stereotypes, prejudice, and discrimination in society (22). Literature indicates that families with mental illness may suffer from more severe stigma, with family members often being blamed as the primary cause of mental illness (23). Stigma not only affects the physical and mental health of parents of adolescents with depression but also indirectly impacts the quality of care received by the adolescents. Studies have found that the higher the mental health literacy and knowledge scores of family members of mentally ill patients, the lower the stigma; and the higher the sense of social support, the lower the stigma (24). This suggests that caregivers can enhance self-acceptance and reduce the harm of stigma by helping parents of adolescents with depression reconstruct their cognition, conduct supportive group interventions, and group self-affirmation training to enhance self-acceptance and reduce the harm of collateralized stigma through health education based on the concept of positive psychology (25).

### Improvement of internal and external family support systems and establishment of a joint school, family, and community care model

The results of this study show that the caregiving burden for parents of adolescents with depression primarily manifests as heavy physical and financial burdens, family dysfunction, and lack of access to professional support information. Positive psychology believes that the process of positive experience and the formation of positive personality also depends on the external environment in which the individual lives. The first level of analysis is the macro level, that is to say, the positive social organization system. The second level is the meso level, that is to say, the positive unit or community organization system. The third and final level is the micro level, which principally refers to the individual’s family organization system (26).

Prolonged and irreplaceable caregiving forces parents to sacrifice much of their disposable time and space, and even lose their original social and economic income. School psychological counseling rooms offer the advantages of convenience, sustainability, and low cost for families of adolescents with depression. This suggests that relevant medical departments should establish psychological and other related medical collaborations with nearby primary and secondary schools, creating a medical model where hospitals and school counseling rooms work together to assist these families. From the meso level, this approach can save parents time accompanying their child to the hospital, reduce some physical burden, lower treatment costs, and alleviate the economic pressure on parents. Meanwhile, the results of this study indicate that adolescent illness is a family stress event, leading to serious parent–child conflicts, couple conflicts, and family relationship imbalances and structural disorders in some families. At the micro level, effective support from family members can significantly improve the quality of life of caregivers, and the more supportive the family is, the more positive the caregiver’s emotional wellbeing is (27). This suggests that caregivers should strengthen their linkage with families of adolescents with depression, guide mutual support between parents, children, and partners, and encourage parents and other relatives to actively communicate their feelings. This approach fosters mutual connection and tolerance, formulates individualized family treatment plans for different conflicts, and creates a family-centered and targeted care intervention model, enabling adolescents to effectively cope with this family stressor. Most caregiving information for mental illnesses cannot be simply replicated from others’ personal experiences. Health education should elaborate on its specific content rather than speaking in generalities, requiring professionals to formulate individualized treatment plans. This suggests that China’s community care should learn from the community psychiatric care models in other countries and establish daycare centers adapted to China’s national conditions. The more social support a caregiver receives, the more likely he or she is to have positive emotions, which can reduce the caregiver’s burden of care and improve his or her ability to cope with stress (28). Hospitals, communities, schools, and families are all involved in the recovery process of the patient’s condition. At the macro level, establishing a joint care model with schools as the bridge, families as the center, and the community as the backbone, supported by healthcare professionals, can alleviate the caregiving burden on parents of adolescents with depression while broadening the channels for obtaining individualized disease care knowledge (29). At the same time, the platform of mutual communication among doctors, nurses and patients can psychologically support parents so that they can think from a positive perspective when facing their own illnesses, adopt proactive behavior to cooperate with treatment and care, and improve their quality of life.

### Tapping into positive emotions to enhance parents’ sense of benefit from illness, as well as individual psychological resilience and family resilience

With the rise of positive psychology, there has been an increased focus on the positive effects of illness - a sense of benefit from the illness. In the case of carers of adolescents with depression, the results of this study show that some parents of adolescents with depression continue to deepen their negative emotions and difficulties, gradually accumulating caregiving burdens; simultaneously, some parents can accumulate their strengths during the caregiving process, experience positive growth, and perceive unexpected gains. It has been demonstrated that caregivers are capable of eliciting positive emotions through active coping with caregiving challenges, enhancement of caregiving abilities, perception of affection between loved ones, and experience of a sense of accomplishment from exerting control (30). In this study, caregivers exhibited positive emotions during the caregiving process and demonstrated positive personality traits, including optimism, courage, and competence. These traits reflect the sense of illness benefit, which is defined as the positive meanings constructed by caregivers in caring for their patients. The same is true of parents’ well-adjusted process of personal psychological resilience in the face of adversity. This finding suggests that healthcare professionals can adopt a positive psychology perspective to enhance the psychological resilience of caregivers, facilitate the construction of benefits derived from illness by caregivers, and facilitate a more profound exploration of their positive emotions and psychological sentiments. Literature suggests that effective regulation of positive emotions not only leads to higher life satisfaction and self-esteem and lower despair and depression but also buffers the effects of negative emotions (31, 32). At the same time, helping carers to explore and demonstrate positive personality traits is significant in promoting their mental health.

Depression, as a psychosomatic illness, tends to have a high cumulative risk of impacting family resilience before and during the illness in adolescents. The findings of the present study demonstrate that some families, subsequent to the experience of depression in their adolescents, have enhanced their familial connections and cultivated more harmonious family relationships. Conversely, other families exhibited a protracted period of dysfunctional family relationships. Family resilience can enable families to gain new strengths and resources in the face of adversity and crisis, thus helping them recover and adapt from adversity (33), and promoting the health of patients and their families (34). Caregivers can help families improve their ability to cope with crisis events by uncovering family resilience in families with depression from a positive psychology perspective (35). Walsh’s family resilience theory divides the core components of family functioning into three coping systems: belief system, organizational patterns, and communication-solving, with multiple key components being intervenable (36, 37). The above reveals that caregivers can start from the perspective of family psychological resilience by identifying family conflicts and strengths in different family coping systems, promoting positive changes in family beliefs, adjusting family organization to closely link family members, guiding self-expression, promoting communication, improving the family’s resilience, coping with family crises together, and reducing parental caregiving burden.

## Limitations

The experiences of parental caregiving for depressed adolescents in this paper were primarily obtained through the 12 interviewers’ review and collation of their own feelings and experiences in the caregiving process. Future research could adopt a longitudinal approach to explore in depth the process of changes in emotional states, coping strategies and caregiving experiences over time, in order to deepen the understanding of parents’ caregiving experiences of depressed adolescents in a more comprehensive way. Future research could also improve the breadth and representativeness of the data by incorporating quantitative research methods to provide additional evidence to support the field.

## Conclusion

In this study, through interviews with 12 parents of adolescents with depression, we found that these parents experienced complex and changing emotional states and faced many dilemmas during the caregiving period. From a positive psychology perspective, some parents of depressed children have demonstrated the power of a positive psyche to enhance individual and family psychological resilience after experiencing stressful family events. This improvement is crucial for reducing the caregiving burden on parents, improving their physical and mental health, and contributing to the patient’s rehabilitation. The role of positive psychology comes to the fore here as it offers new ideas and approaches for caregivers and medical social workers. Nurses and medical social workers should develop models of care based on individual and family psychological resilience and sense of benefit from illness through the lens of positive psychology, with schools as the bridge, families as the center, and the community as the foundation, to promote the rehabilitation of patients and their families.

## Data Availability

The raw data supporting the conclusions of this article will be available from the corresponding author on reasonable request.
